# Dosimetric assessment of an air‐filled balloon applicator in HDR vaginal cuff brachytherapy using the Monte Carlo method

**DOI:** 10.1002/acm2.12298

**Published:** 2018-03-01

**Authors:** Hongyu Jiang, Rajeev Badkul, Damodar Pokhrel

**Affiliations:** ^1^ Department of Radiation Oncology The University of Kansas Cancer Center Kansas City KS USA

**Keywords:** brachytherapy, Capri applicator, HDR, Monte Carlo

## Abstract

**Purpose:**

As an alternative to cylindrical applicators, air‐inflated balloon applicators have been introduced into high‐dose‐rate (HDR) vaginal cuff brachytherapy to achieve sufficient dose to the vagina mucosa as well as to spare organs at risk, mainly the rectum and bladder. Commercial treatment planning systems which employ formulae in the AAPM Task Group No. 43 (TG 43) report do not take into account tissue inhomogeneity. Consequently, the low‐density air in a balloon applicator induces different doses delivered to the mucosa from planned by these planning systems. In this study, we investigated the dosimetric effects of the air in a balloon applicator using the Monte Carlo (MC) method.

**Methods:**

The thirteen‐catheter Capri™ applicator by Varian™ for vaginal cuff brachytherapy was modeled together with the Ir‐192 radioactive source for the microSelectron™ Digital (HDR‐V3) afterloader by Elekta™ using the MCNP MC code. The validity of charged particle equilibrium (CPE) with an air balloon present was evaluated by comparing the kerma and the absorbed dose at various distances from the applicator surface. By comparing MC results with and without air cavity present, dosimetric effects of the air cavity were studied. Clinical patient cases with optimized multiple Ir‐192 source dwell positions were also explored. Four treatment plans by the Oncentra Brachy™ treatment planning system were re‐calculated with MCNP.

**Results:**

CPE fails in the vicinity of the air‐water interface. One millimeter beyond the air‐water boundary the kerma and the absorbed dose are equal (0.2% difference), regardless of air cavity dimensions or iridium source locations in the balloon. The air cavity results in dose increase, due to less photon absorption in the air than in water or solid materials. The extent of the increase depends on the diameter of the air balloon. The average increment is 3.8%, 4.5% and 5.3% for 3.0, 3.5, and 4.0 cm applicators, respectively. In patient cases, the dose to the mucosa is also increased with the air cavity present. The point dose difference between Oncentra Brachy and MC at 5 mm prescription depth is 8% at most and 5% on average.

**Conclusions:**

Except in the vicinity of the air‐mucosa interface, the dosimetric difference is not significant enough to mandate tissue inhomogeneity correction in HDR treatment planning.

## 1 INTRODUCTION

In high‐dose‐rate (HDR) brachytherapy, single‐catheter solid cylinder applicators have been accepted to be the standard care for post‐operative vaginal vault treatment to reduce the recurrence risk. According to a survey by the American Brachytherapy Society (ABS), a single channel solid cylinder was utilized by more than 90% of the respondents.^1^ This type of applicators provides simplicity in applicator insertion, treatment planning, and radiation delivery. However, these simple devices also come with two major deficiencies. First of all, in axial views (perpendicular to applicator central axis), the isodose curves are in circular shapes. Delivery of a sufficient dose to the vaginal mucosa is usually accompanied by undesired high doses to the anterior rectal wall and/or the posterior bladder. Multi‐channel solid applicators such as the Miami‐style applicators (seven‐channel applicator originally developed at the Sylvester Cancer Center in Miami, Florida) and the VCMC™ (Vaginal CT/MR Multi Channel) applicator by Elekta have been introduced to serve as an alternative to improve the dose distributions. Via dwelling the radioactive source in peripheral channels, a non‐circular and more conformal dose distribution to the mucosa may be achievable. A second major issue with solid applicators (single and multiple channel ones) has to do with the potential presence of air gaps between the applicator and the mucosa after applicator insertion. To deliver a sufficient dose to the vaginal mucosa, a direct contact of the applicator outer surface with the vaginal mucosa is imperative,as reported by the ABS.^2^ Even a small gap of 1–2 mms can result in a significant dose reduction to the mucosa. As noticed by our and other institutions,^3^, ^4^ it is not uncommon to notice from patient CT scans the presence of air gaps at the apex of or along a solid applicator, as long as the vaginal cavity is not in a perfect cylindrical shape after applicator insertion.

The introduction of multi‐catheter air balloon applicators is intended to resolve both issues associated with solid applicators. With multiple catheters, the rectum and bladder are spared without coverage sacrifice thanks to source dwelling in lateral catheters. Meanwhile, if the outer layer of the balloon applicator presents sufficient elasticity, the shape of the vaginal cavity would not pose a major issue. Pressure from the injected air or saline will push the balloon surface to firmly contact the vaginal wall. Our patient study has confirmed the efficacy of the balloon style Capri vaginal applicator in removing the air gaps around the applicator surface.^5^ We randomly selected 25 patients treated with the Capri applicator and another 25 patients treated with the single‐catheter cylinder applicators. Among the patients treated with the cylinder applicators, ring‐type air gaps (due to a smaller size of cylinder in the vagina) appeared in 12 patient CT scans. As a comparison, ring‐type air gaps appeared in none of the patients treated with the Capri applicator.

One concern regarding air‐inflated applicators is the accuracy of dosimetry. We use the program of Oncentra Brachy™ (version 4.5.2) by Elekta for HDR treatment planning. Similar to other treatment planning systems based on the TG 43 formulae,^6^ in Oncentra Brachy all types of tissue are treated as water equivalent without any tissue inhomogeneity correction. Replacement of the air inside the balloon with water in dose calculations results in an overestimate of the attenuation of primary photons and an underestimate of the dose of the scattered electrons from the applicator. The actual delivered dose may deviate from what's reported by Oncentra Brachy. Although in HDR brachytherapy, the distance accuracy plays a more dominant role than the tissue composition, it has been a consistent concern of our physicians that a clinically significant dose variation might be present with balloon applicators. Research on the SAVI™ partial breast air balloon applicator by Cianna Medical™ showed a dosimetric effect in the range of 3–9%.^7^ The primary goal of this study is to evaluate the dosimetric effect of the air cavity in the Capri balloon applicator using the Monte Carlo (MC) method with the MCNP code.

## 2 MATERIALS AND METHODS

### 2.A. The Multi‐catheter Capri balloon applicator

**Figure 1:**
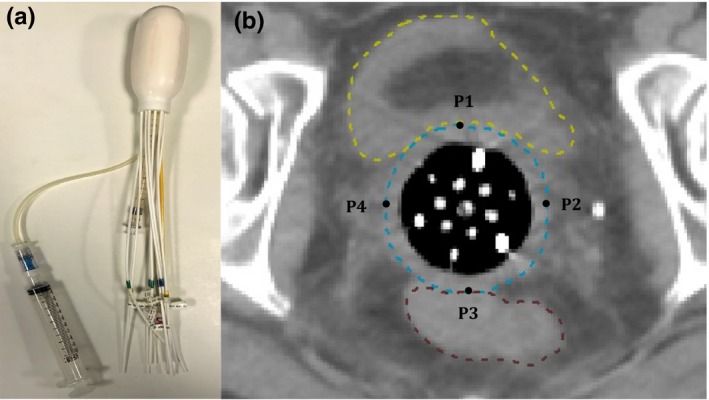
Introduction of the Capri applicator: (a) picture of a 13‐catheter Capri applicator with a syringe connected; (b) an axial CT image of a Capri applicator in patient. Contours include the bladder, the rectum, and the envelope of Capri with 5 mm expansion. Prescription points (P1–P4) placed onto the envelope are used in plan optimization. There are a total of 16 prescription points (P1–P16) for each MC re‐calculated plan.

The Capri applicator was invented by Dr. Gail Lebovic, and was the most recent addition to the Varian's applicator inventory. The applicator is intended for brachytherapy treatment of vaginal and rectal diseases. The inflatable design makes the applicator flexible, accommodative, and comfortable to the potential patients. The applicator contains 13 lumens arranged in two concentric rings (six lumens each ring) surrounding a central lumen, as shown in Fig. 1. Markers of different length are attached to the lumens numbered 2, 4, and 6 to allow for easy identification of them in CT images. According to the manufacturer specifications, the deflated insertion diameter is 2.9 cm. Allowance of variable fill volume makes Capri a one‐size‐fits‐all applicator. The balloon diameter after inflation was in the range of 3.5–4.1 cm for about 30 patients treated in our institution so far. The balloon wall is made of soft silicone, and its thickness is about 0.5 mm when deflated. The balloon can be inflated with either saline or air. Saline was tried in our clinic for the first several cases to achieve water equivalent. However, our physician realized that it was challenging to fill up the applicator interior thoroughly, and for physicists it was difficult to recognize the catheters in CT images in saline‐filled regions during treatment planning. Later on, a decision was made to adopt air instead.

### 2.B. Ir‐192 source modeling

The Nucletron microSelectron HDR Iridium‐192 (Ir‐192) source was modeled using the MC N‐Particle transport code MCNP version 6.0 by Los Alamos National Laboratory.^8^ The geometry of the Ir‐192 source was illustrated and described in great details by others.^9^ The energy distribution of Ir‐192 photons has also been well summarized.^10^ Photons of Bremsstrahlung, electron capture, and *β* ^‐^ decay were all included in our MC simulations. To benchmark the correctness of our iridium source modeling, calculations of the TG 43 parameters adopted into Oncentra Brachy^9^ were repeated, and excellent agreements were achieved. The discrepancy of dose rate per unit air‐kerma strength (cGyh^−1^ U^−1^ in table III in reference^9^) was generally within 2%. At the longest distances from the source (lowest dose regions), the relative difference might be over 2%, but the absolute difference was insignificant (<0.005 cGyh^−1^ U^−1^).

### 2.C. Validity of charged particle equilibrium

Charged particle equilibrium (CPE) holds well for HDR with solid cylindrical applicators, i.e. the absorbed dose (energy deposited per unit mass) and the kerma (energy transferred from photons per unit mass) are identical in value. After the introduction of a large air cavity surrounding the HDR source, CPE is still expected to be valid at locations distant from the applicator. However, at the vicinity of the balloon surface (air‐tissue interface), the prevalence of CPE may be susceptible. Without CPE, the TG 43 formulae may not be suitable, and the planned dose by Oncentra Brachy may not be realistic, either. Since the vaginal mucosa is located within millimeters from the applicator surface, the validity of CPE should be examined.

One additional benefit of CPE presence is the unnecessity of transporting electrons in MC simulations, i.e., the kinetic energy transferred from photons to electrons can be treated as locally deposited. Tracking electrons is very time consuming, and photon‐only MC simulations are known to be much faster than photon‐electron coupled ones.

In our MC model, an air‐filled cylindrical applicator of 3.0, 3.5, or 4.0 cm in diameter was placed in infinite water. One iridium source was positioned either at the applicator's geometrical center, or at off‐center sites where the lumens of two concentric rings of Capri were located. The diameters of the applicator were chosen to cover the range of balloon sizes in patient care: a minimum of 2.9 cm when deflated, and a maximum of 4.1 cm as observed in our patient cases. Eighteen tally spheres of 0.1 mm in diameter were placed in water, ranging from 0.2 to 70 mm from the applicator surface to the tally sphere center (0.2, 0.4, 0.6, 0.8, 1, 1.2, 1.5, 2.5, 3.5, 5, 7.5, 10, 15, 20, 25, 30, 50, and 70 mm). Secondary electrons were transported down to a cutoff energy of 1 keV. The absorbed dose (*F8:P) and the kerma (F6:P) were collected for each of the 18 tally spheres. Comparison of the absorbed dose to the kerma indicated CPE validity at these preselected locations.

### 2.D Dosimetric effects of the air cavity

By comparison of doses with or without the air cavity under the same iridium source condition, the impact of the air cavity on HDR dosimetry can be evaluated. An additional set of MC calculations were performed with the same models as in Section 2.A.C, except that the air cavity was replaced with water.

### 2.E Patient study

About 30 patients who suffered intravaginal diseases had been treated with Capri in our institution. The procedure of treatment planning was the following. After a Capri applicator was inserted and inflated with air, the patient was scanned with 2 mm slice thickness on a Philips™ Brilliance Big Bore™ 16‐slice CT scanner. The CT images were imported into Oncentra Brachy, and organs at risk (the bladder, the rectum, the sigmoid, and the small bowel) were contoured by the physicians, following the RTOG (Radiation Therapy Oncology Group) guidelines (available at https://www.rtog.org/corelab/ContouringAtlases.aspx). The radiation dose was prescribed at 5 mm depth from the applicator surface. The applicator envelope was contoured, and then expanded by 5 mm as CTV (clinical target volume). Next, the dose points (usually 16 dose points for covering 4 cm of treatment length) were placed onto the CTV outlines. Thirteen catheters were reconstructed in the order of catheter 1 to catheter 13. With a source step size of 5 mm, the dwell positions of the Ir‐192 source in each catheter were activated to cover the top 4 cm of the mucosa from the applicator tip. The *Points* optimization tool in Oncentra Brachy was then utilized to optimize the dwell time at the activated dwell positions to achieve the prescription dose at the CTV dose points. The point optimization process did not take into account of the rectum and bladder. In most cases, the rectal and bladder doses would exceed our physician's demand. Using the *Graphical* optimization tool, the isodose curves were visually moved closer to the balloon applicator in the posterior (rectal) and anterior (bladder) directions to lower the maximum rectal and bladder dose to 70% and 80% of the prescription dose, respectively. At the apex, the isodose curves might also be adjusted to spare the sigmoid and small bowel as needed. Because the nearest applicator‐rectum or applicator–bladder distance might be <5 mm, as a consequence of rectum and bladder sparing, the dose of some CTV dose points on the bladder or rectal side would be lower or even considerably lower than the prescription dose. As the advantage of the multi‐catheter applicators, little coverage loss on the right and left sides of the applicator happened during graphical optimization. Sixteen dose points were chosen per plan for quantitative comparison between Oncentra Brachy and MC. As shown in Fig. 1(b), four points (anterior, posterior, left, and right) were chosen per axial CT slice. A total of four CT slices were chosen with 1 cm spacing. Sixteen points in four CT slices of 1 cm spacing would therefore cover 4 cm, matching the prescribed treatment length.

After computerized optimization and manual adjustment, a treatment plan would include a total of 70–80 non‐zero dwell positions. It would be cumbersome and very time consuming if each source dwell position in a treatment plan required one lengthy MC simulation to be run. Therefore a simpler scheme of creating a data library was adopted instead. MC calculations for combinations of various sizes of air cavities (immerged in infinite water) and source locations in air cavities were completed. The dose distributions of these calculations were stored in a library. When re‐calculating an Oncentra plan with MC, the dose of a CTV dose point was obtained by adding up the contribution from each dwell position in the plan, which was acquired from the data library. This simplified approach did not differentiate the soft tissue and the bone tissue, i.e., all tissue types were treated as water equivalent. Compared to the air cavity, the dosimetric effect of tissue differentiation was expected to be much smaller.

The diameter of a deflated Capri applicator is 2.9 cm. The diameter of an inflated Capri in patients whom we treated was in a narrow range of 3.4–4.1 cm. It became unnecessary to include a large number of patient samples. As a result, only four cases were selected in this study: two cases on the small diameter side (3.5 cm) and two cases on the large diameter side (4.0 cm).

## 3 RESULTS

### 3.A CPE

**Figure 2:**
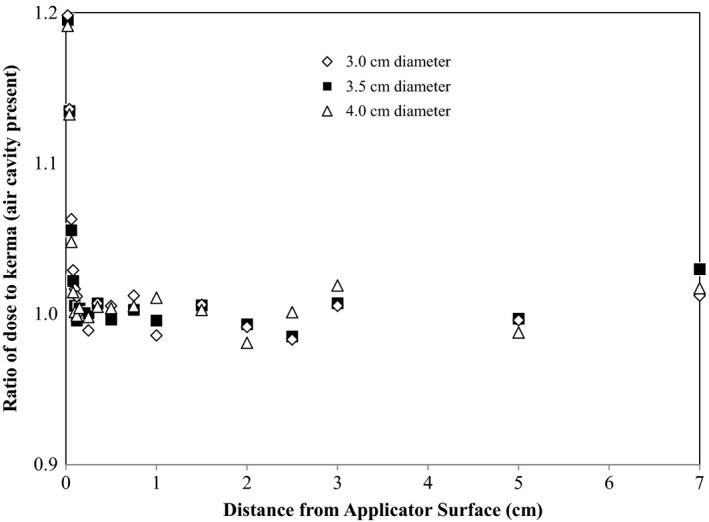
Ratio of the absorbed dose to the kerma calculated with Monte Carlo for an air‐inflated cylindrical balloon of three sizes (3, 3.5, and 4.0 cm in diameter), with the Ir‐192 seed placed at the center of the balloon.

As plotted in Fig. 2, within 1 mm from the air‐water interface, the absorbed dose can be significantly higher than the kerma. For example, at 0.2 mm depth from the applicator, the absorbed dose is approximately 20% more than the kerma. Beyond 1 mm, on the other hand, the two are basically identical, with the absorbed dose on average 0.2% more than the kerma, independent of the balloon size. Figure 2 shows the results for a centrally positioned iridium source. The same conclusion was drawn from MC simulations with off‐center source dwell positions, i.e., from 1 mm and beyond, there is no noticeable difference between the absorbed dose and the kerma. Therefore, except in the vicinity of the balloon applicator envelope, CPE can be deemed existent in water or tissue. For improving computational speed, the secondary electrons need not to be transported in MC simulations if there is no specific interest in radiation doses within 1 mm from the applicator.

The cause of dose increase near the applicator surface was studied further. It was discovered that the extra energy deposition was mainly from the scattered or secondary electrons. Inside the air cavity, two major sources of secondary electrons exist: the electrons emitted from the Ir‐192 source capsule, and the electrons generated in water via photon interactions and then scattered back to the balloon applicator. In the case of a solid cylindrical applicator, all electrons from the Ir‐192 source and a significant fraction of the backscattered electrons deposit their entire energy and are absorbed inside the applicator body. However, with a balloon applicator, due to little attenuation of electrons in the air, these electrons travel through the applicator cavity and reach to the tissue contacting the applicator envelope. Results from MC simulations showed an increase in electron fluence of over 30% at the applicator‐water interface. The kinetic energy of the majority (>90%) of these electrons is below 0.3 MeV, corresponding to a range of 1 mm in water. The dose gradient in the first millimeter of water is very steep. Beyond 1 mm, after most scattered electrons are absorbed and the boost to absorbed dose disappears, the absorbed dose and the kerma start to show consistent agreement.

### 3.B Air cavity dosimetric effects

**Figure 3:**
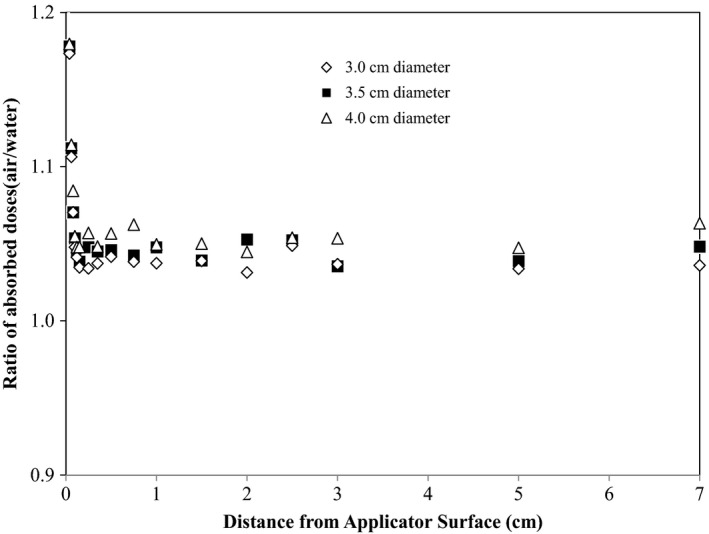
Ratio of the absorbed dose with the air cavity present to the absorbed dose with the air cavity absent (water) calculated with Monte Carlo for a cylindrical balloon of three sizes (3, 3.5, and 4.0 cm in diameter), with the Ir‐192 seed placed at the center of the balloon.

Figure 3 shows the ratio of the absorbed doses with and without the air cavity for the case of a single Ir‐192 source at the applicator center. Similarly, with the air cavity present, the absorbed dose in the first 1 mm from the applicator surface can be much higher. After the scattered electrons from the applicator are absorbed, the absorbed dose drops to a level only a few percent higher than the dose with air cavity filled with homogeneous water. The air cavity induces dose increase in the region of CPE presence (>1 mm), and the extent of increase depends on the diameter of the air cavity. The average increment is 3.8%, 4.5% and 5.3% for 3.0, 3.5, and 4.0 cm applicators, respectively. The dose increase is mainly attributed to less absorption of the Ir‐192 photons in the air cavity than in water or solid applicator bodies. The energy absorption coefficient *μ*_en_/*ρ* of 0.34 MeV photons in water is 0.032 cm^2^/g, corresponding to an average loss of several percent of photon energy in the air cavity of the size of a normal balloon applicator.

For off‐center or peripheral iridium source dwell positions, as expected, the effect to the absorbed dose depends on not only the size of the air cavity, but also the location of the Ir‐192 source in a balloon applicator. At an off‐center source dwell position, photons from the iridium source travel different lengths in the air cavity in different directions. In the direction, the photons travel the longest distance in the air, the dosimetric effect is the greatest, and vice versa. Extensive MC calculations were performed for the Capri applicator. Two applicator sizes were chosen: 35 and 40 mm. A modeled iridium source was placed at either the inner or outer concentric ring of the applicator. The followings are the results for the maximum (MAX, corresponding to longest air cavity traveling length) and minimum (MIN, corresponding to shortest air cavity traveling length) dose increase: for 40 mm applicator size, outer ring MAX 9.6%, outer ring MIN 0.84%, inner ring MAX 7.6%, inner ring MIN 2.9%; for 35 mm applicator size, outer ring MAX 8.4%, outer ring MIN 0.5%, inner ring MAX 6.8%, inner ring MIN 2.2%. So the maximum effect from a single dwell position is in the range of 8–9%. The overall effect upon a clinical treatment plan containing multiple dwells will be the weighted average percent increase in individual dwell positions.

### 3.C Patient study results

All the dose points in the patient study were placed at 5 mm depth from the applicator surface, and therefore no electrons were transported in MC simulations. All electrons were terminated after generation and their energy was locally deposited and recorded for dose accumulations. The calculated doses for 16 dose points of each case are presented in Tables 1 and 2 for 35 and 40 mm applicators, respectively. The point doses from Oncentra Brachy and the dose percent differences between Oncentra Brachy and MC are shown in Tables 1 and 2 as well, providing a comparison between the air cavity case (MC) and the water‐equivalent case (Oncentra Brachy) under the same treatment plan. According to Tables 1 and 2, the doses of all points go higher when the air in the cavity is modeled in dose calculations. As stated in the preceding section, the dosimetric effect to different dose points differs. For a dose point, the effect of the air cavity depends on the dwell pattern of the iridium source in the treatment plan. If a dose point is overall far away from heavily loaded catheters, the dosimetric effect to this point will be greater, and vice versa. From Tables 1 and 2, the maximum point dose difference is <8%, and on average Oncentra Brachy underestimates the dose by 5%. Furthermore, the effect of balloon size (35 mm vs. 40 mm) is insignificant for these four patient cases.

**Table 1:** Comparison between doses (cGy) calculated with Oncentra (air in balloon treated as water) and Monte Carlo (air in balloon modeled as air) for two patient cases of 35 mm Capri applicator

35 mm diameter case 1				35 mm diameter case 2			
Point #	TPS dose	MC dose	% difference	Point #	TPS dose	MC dose	% difference
1	441.6	462.7	4.8	1	395.3	411.2	4.7
2	483.6	500.9	3.6	2	476.9	496.0	4.0
3	334.7	354.9	6.1	3	291.3	311.7	7.0
4	479.8	498.2	3.8	4	479.1	496.6	3.7
5	423.5	444.6	5.0	5	403.0	420.6	4.4
6	499.0	516.1	3.4	6	498.4	517.2	3.8
7	358.7	380.8	6.2	7	302.8	323.2	6.7
8	512.7	533.3	4.0	8	536.0	553.2	3.2
9	424.6	446.2	5.1	9	390.4	407.2	4.3
10	521.5	538.0	3.2	10	510.2	529.1	3.7
11	365.9	389.1	6.3	11	293.6	314.2	7.0
12	535.9	556.6	3.9	12	540.1	556.5	3.0
13	408.2	429.9	5.3	13	387.5	405.1	4.6
14	481.9	496.2	3.0	14	437.3	455.0	4.1
15	331.8	354.3	6.8	15	285.0	307.2	7.8
16	480.0	499.3	4.0	16	445.9	459.6	3.1

**Table 2:** Comparison between doses (cGy) calculated with Oncentra (air in balloon treated as water) and Monte Carlo (air in balloon modeled as air) for two patient cases of 40 mm Capri applicator

40 mm diameter case 1				40 mm diameter case 2			
Point #	TPS dose	MC dose	% difference	Point #	TPS dose	MC dose	% difference
1	408.7	429.1	5.0	1	405.1	425.0	4.9
2	512.2	533.0	4.1	2	444.8	460.7	3.6
3	415.7	435.9	4.9	3	346.5	368.7	6.4
4	490.3	509.8	4.0	4	452.5	478.2	5.7
5	410.8	432.4	5.3	5	446.6	466.7	4.5
6	519.7	539.4	3.8	6	522.9	538.7	3.0
7	454.6	475.3	4.6	7	356.6	379.0	6.3
8	505.8	524.7	3.7	8	510.6	537.0	5.2
9	397.4	417.9	5.2	9	463.1	482.2	4.1
10	483.1	501.2	3.8	10	530.7	544.3	2.6
11	450.3	470.3	4.5	11	355.6	379.0	6.6
12	496.2	514.8	3.8	12	528.4	555.1	5.1
13	398.3	420.8	5.7	13	442.4	462.0	4.4
14	461.2	482.5	4.6	14	480.6	493.5	2.7
15	447.0	470.8	5.3	15	360.4	386.0	7.3
16	464.2	486.3	4.8	16	494.4	522.5	5.7

In external beam radiotherapy with X rays, 5% of dose uncertainty is clinically meaningful. However, in brachytherapy with radioactive sources, the distance from the sources plays a much more dominant role, and the dose gradient is usually much higher than that in external beam radiotherapy. Five percent of dose discrepancy can be equivalently introduced by a positioning uncertainty of <1 mm, which is within the acceptable tolerance in brachytherapy. As for at the very vicinity of balloon surface (<1 mm), although not studied in patient cases, significantly higher doses than Oncentra Brachy could be expected.

## 4 DISCUSSIONS

With the application of an air balloon applicator, CPE fails at the very proximity of air/tissue interface (<1 mm). The dose to the mucosa could be notably higher than reported by treatment planning programs. Within this 1 mm region, due to the steepness of the dose gradient, it might be challenging to accurately estimate the dose. Beyond 1 mm, the dose to the mucosa is only slightly elevated (3.8–5.3% on average for the case of a single source located at the center of the applicator), which is attributed to slightly less photon attenuation in the air than in an solid applicator. Our patient study did not discover significant dosimetric differences (8% at maximum and 5% on average) at 5 mm prescription depth between air‐filled and water‐equivalent applicators, either. Treatment planning with Oncentra Brachy or other TG‐43 style planning systems is clinically acceptable for the air balloon applicators such as the Capri.

In HDR vaginal cuff brachytherapy, two methods of prescribing radiation dose was suggested by the ABS^11^: dose is prescribed to either the vaginal surface or at 5 mm depth. With balloon applicators, prescribing dose to the applicator surface may not be applicable anymore, because doses at the applicator surface in treatment plans might not be realistic. On the other hand, the dose at 5 mm depth is insignificantly affected by air balloon, and as a result the use of 5 mm as the prescription depth is still feasible.

## 5 CONCLUSIONS

Tissue inhomogeneity is not considered in dose calculations based on the AAPM TG43 algorithms. The use of the balloon style applicators in partial breast or gynecologic HDR treatment incurs concerns about the impact of a large air volume on dose calculation accuracies. It was demonstrated in our study of the Capri applicator that within 1 mm from the applicator surface, a significant higher dose than reported by HDR treatment plans would occur. Accordingly, the approach to prescribe a radiation dose to the applicator surface may not be appropriate any more. In our patient case studies, the radiation dose was prescribed at 5 mm depth. At this depth, the dose discrepancy between MC and TG 43 algorithm was 5% on average, which is considered clinically acceptable for HDR brachytherapy. Therefore, inhomogeneity correction for the air inside a balloon applicator may not be needed.

## CONFLICT OF INTEREST

The authors have no conflicts of interest to declare.
